# Medical imaging in personalised medicine: a white paper of the research committee of the European Society of Radiology (ESR)

**DOI:** 10.1007/s13244-015-0394-0

**Published:** 2015-03-13

**Authors:** European Society of Radiology (ESR)

**Affiliations:** Neutorgasse 9/2, 1010 Vienna, Austria

**Keywords:** Individualized medicine, Theranostics, Biomarkers, Radiogenomics, Precision medicine

## Abstract

The future of medicine lies in early diagnosis and individually tailored treatments, a concept that has been designated ‘personalised medicine’ (PM), which aims to deliver the right treatment to the right patient at the right time. Medical imaging has always been personalised and is fundamental to almost all aspects of PM. It is instrumental in solving clinical differential diagnoses. Imaging procedures are tailored to the clinical problem and patient characteristics. Screening for preclinical disease is done with imaging. Stratification based on imaging biomarkers can help identify individuals suited for preventive intervention. Treatment decisions are based on the in vivo visualisation of the location and extent of an abnormality, as well as the loco-regional physiological, biochemical and biological processes using structural and molecular imaging. Image-guided biopsy provides relevant tissue specimens for genetic/molecular characterisation. In addition, radiogenomics relate imaging biomarkers to these genetic and molecular features. Furthermore, imaging is essential to patient-tailored therapy planning, therapy monitoring and follow-up of disease, as well as targeting non-invasive or minimally invasive treatments, especially with the rise of theranostics. Radiologists need to be prepared for this new paradigm as it will mean changes in training, clinical practice and in research.

*Key Points*

• *Medical imaging is a key component in personalised medicine*

• *Personalised prevention will rely on image-based screening programmes*

• *Anatomical, functional and molecular imaging biomarkers affect decisions on the type and intensity of treatment*

• *Treatment response assessment with imaging will improve personalised treatment*

• *Image-based invasive intervention integrates personalised diagnosis and personalised treatment*

## Introduction

In 2011, the ESR published its first white paper on personalised medicine (PM) [1]. The focus of this article was on the important and essential role of medical imaging in early diagnosis and individually tailored treatment. The article concluded that medical imaging plays a critical role in all aspects of PM: prediction, diagnosis and especially treatment. Thus, for PM to reach its highest potential, medical imaging must play an integral role. As the developments in PM are rapid, it is time to update the original white paper and provide a thorough overview of all areas in PM in which imaging plays a role. Currently, national and European funding agencies as well as public-private collaborations support large research programmes and infrastructures dedicated to PM [2]. It is important that the benefits and contributions of clinical imaging and imaging research to PM are acknowledged and supported in order to fulfil the promises of PM. As PM has already entered the clinical arena, it is also important that the medical imaging community is aware of this concept and is prepared for participating as a relevant partner in PM.

Therefore, the ESR considers it of strategic importance to describe how medical imaging contributes to PM and to delineate major obstacles to the full application of imaging in PM in daily practice. This white paper concludes with recommendations for the medical imaging field.

### Definition

Sequencing of the human genome in 2003 opened the way to identifying specific genes that are involved in particular diseases and thus supports the identification of genetic variants that either predispose an individual to a specific disease or regulate that individual’s sensitivity to a given treatment [3]. The promise of PM lies in the prediction of disease risk, the choice of the right treatment and the assessment of the treatment response and safety profile based on genomic sequence data. Thus, many consider PM to be synonymous with genomic medicine (Table 1). However, the European Science Foundation (ESF) stated that the range of information that can be used to customise healthcare is far greater than genome sequence data alone. Their definition of PM is broadly described as a customisation of healthcare that accommodates individual differences as far as possible at all stages in the process, from prevention, through diagnosis and treatment, to post-treatment follow-up. In other words, it is about providing the right treatment to the right patient at the right time [3]. The National Academy of Sciences of the USA prefers the term “precision medicine” instead of “personalised medicine” as it does not literally mean the creation of drugs or medical devices that are unique to a patient, but rather the ability to classify individuals into subpopulations that differ in their susceptibility to a particular disease, in the biology and/or prognosis of those diseases they may develop, or in their response to a specific treatment [4].

**Table 1:** Definitions of terms related to personalised medicine

Personalised medicine
• According to Wikipedia, "Personalized medicine is a medical model that proposes the customization of healthcare using molecular analysis—with medical decisions, practices, and/or products being tailored to the individual patient. In this model, diagnostic testing is often employed for selecting appropriate and optimal therapies based on the context of a patient’s genetic content. The use of genetic information has played a major role in certain aspects of personalised medicine and the term was first coined in the context of genetics, though it has since broadened to encompass all sorts of personalization measures" [104]
• The US National Human Genome Research Institute defines personalised medicine as an emerging practice of medicine that uses an individual’s genetic profile to guide decisions made in regard to the prevention, diagnosis and treatment of disease. Knowledge of a patient’s genetic profile can help doctors select the proper medication or therapy and administer it using the proper dose or regimen [105]
• The US National Cancer institute describes personalised medicine as a form of medicine that uses information about a person’s genes, proteins, and environment to prevent, diagnose, and treat disease. In cancer, personalised medicine uses specific information about a person’s tumour to help diagnose, plan treatment, find out how well treatment is working, or make a prognosis. Also called precision medicine [106]
• European Science Foundation: Personalised medicine can be broadly described as a customisation of healthcare that accommodates individual differences as far as possible at all stages in the process, from prevention, through diagnosis and treatment, to post-treatment follow-up [3]
• National Academy of Sciences: Precision medicine refers to the tailoring of medical treatment to the individual characteristics of each patient. It does not literally mean the creation of drugs or medical devices that are unique to a patient, but rather the ability to classify individuals into subpopulations that differ in their susceptibility to a particular disease, in the biology and/or prognosis of those diseases they may develop, or in their response to a specific treatment. Preventive or therapeutic interventions can then be concentrated on those who will benefit, sparing expense and side effects for those who will not. Although the term “personalised medicine” is also used to convey this meaning, that term is sometimes misinterpreted as implying that unique treatments can be designed for each individual. For this reason, the Committee thinks that the term “precision medicine” is preferable to “personalised medicine” [4]
Pharmacogenomics
The study of how a person’s genes affect the way he or she responds to drugs. Pharmacogenetics is being used to learn ahead of time what the best drug or the best dose of a drug will be for a person [106]
Genomic medicine
The use of genetic information to improve health outcomes
Stratified medicine
The identification of subgroups of patients with a particular disease who respond to a particular drug or, alternatively, are at risk of side effects in response to a certain treatment [3]
Theranostics
The term “theranostics” was coined to define ongoing efforts in clinics to develop more specific, individualised therapies for various diseases and to combine diagnostic and therapeutic capabilities into a single agent [107]
Radiogenomics
Radiogenomics is a relatively recently coined term that describes the relationship between imaging features of a lesion and the underlying genetic/molecular features. It can be helpful for improved personalised diagnosis, prognosis, and assessment of treatment response [72]
Companion diagnostics
A companion diagnostic device can be an in-vitro diagnostic device (IVD) or an imaging tool that provides information that is essential for the safe and effective use of a corresponding therapeutic product. The use of an IVD companion diagnostic device with a particular therapeutic product is stipulated in the instructions for use in the labelling of both the diagnostic device and the corresponding therapeutic product as well as in the labelling of any generic equivalents and biosimilar equivalents of the therapeutic product [108]
Genomic profile
Information about all the genes in an organism, including variations, gene expression, and the way those genes interact with each other and with the environment. A genomic profile may be used to discover why some people get certain diseases while other people do not or why people respond differently to the same drug [106]

Genetic differences may determine disease predisposition and therapeutic response in some diseases. However, most diseases are polygenic in nature and highly influenced by environmental factors including climate, social context and behavioural factors such as diet and exercise. The final disease manifestation or phenotype is the result of gene-environment interaction, and it is expected that differences in the biochemical, biological and physiological characteristics (e.g., perfusion, flow, metabolism, diffusion) of diseased lesions are important factors to be taken into account when tailoring personalised treatment. For that reason the ESF stated that “genomics, epigenomics, proteomics, metabolomics, lipidomics and other ‘omics technologies, such as analysis of the microbiome, will be required alongside imaging and physiological monitoring to generate biological data. All of this information, along with additional data on environmental exposures and lifestyle, for instance, will need to be integrated, analysed and interpreted” [3].

PM is not only relevant for diagnosis and treatment of cancer, but for most persistent diseases that require aggressive treatment or treatment with considerable side effects. PM not only takes into account disease but also patient characteristics as these may also influence the susceptibility to treatment and side effects [5]. PM is not a totally new approach. Medical care is traditionally delivered at a personal level, and clinicians adapt their therapeutic approach to the individual needs of the patient. In this unique physician-patient relationship, all relevant aspects of a human being and its context are taken into account in both the diagnostic process as well as the subsequent treatment, given the state-of-the-art knowledge at the time of the physician-patient interaction. In the past, personal experience has been the prevailing source of knowledge whilst more recently this knowledge has been amended, sometimes to a greater extent, by the collective experience recorded in scientific publications. This has led to specific guidelines, which are based on diagnostic and treatment outcome in a large number of patients with the same type of disease. PM as perceived today will take into account more in-depth diagnostic information from the patient and its disease for the right treatment allocation.

The promises of PM are better diagnoses, earlier interventions, more efficient drug development, more effective therapies, reduction of side effects of treatment and improved cost effectiveness. Better diagnosis is based on information on genetic variants, gene expression, and biological and physiological description of disease. Earlier intervention is based on better diagnostic tools to identify individuals with a predisposition for the development of a particular condition and to monitor interventions that might prevent it, delay its onset or reduce its impact. More efficient drug development is based on a better understanding of disease, which could help scientists identify new disease subgroups and their associated molecular pathways as well as design drugs that target them. More effective therapies are achieved by selecting the right patients for treatment and by selecting the type of treatment (surgery, radiotherapy, chemotherapy and/or targeted therapy) and the intensity of treatment (the best dosing schedule or radiation dose for a particular patient). Reduction of side effects of treatment is achieved by identification of patients who are at risk for developing side effects, by reduction of the number of patients that are exposed to non-effective treatment. Cost-effectiveness is achieved by pre-selecting patients for treatment or by terminating inefficient treatments early.

There are multiple areas in which medical imaging has and will have a major impact in PM (Table 2).

**Table 2:** Imaging and personalised medicine

Diagnosis:
From symptoms to underlying diagnosis with the support of imaging
Diagnostic procedure:
Imaging procedures tailored to the clinical problem and patient characteristics
Prevention/screening:
Imaging to improve risk stratification or to detect preclinical disease
Selection of the right treatment with imaging information
Imaging to assess the localisation and extent disease
Imaging for functional and biological characterisation of lesions
Image-guided biopsy to get specimens for genetic/molecular characterisation
Imaging biomarkers to predict response to treatment, recurrence and survival
Agents that can be used for imaging diagnostics and therapeutic purposes (theranostics)
Imaging biomarkers associated with genetic/molecular features (radiogenomics)
Evaluation of treatment response:
Imaging to assess early the effects of treatment in order to adapt treatment
Imaging in drug discovery and drug trials:
Surrogate imaging biomarkers to evaluate the effect of new personalised treatment
Personalised treatment:
Image-guided treatment with surgical, radiotherapeutical, minimally or non-invasive interventional procedures

## Personalised diagnosis

Medical imaging intrinsically enables PM. The first step leading from clinical symptoms and signs to a diagnosis relies on imaging in a substantial number of diseases. Besides imaging, laboratory analysis of body fluids such as blood, serum, urine or stool also play an important role in the diagnostic process. Imaging has played this role for decades by the assessment of the location of disease in individual patients and by characterisation of the structural abnormalities. While classical macroscopic imaging provides some of the most important ‘individualised’ information on many diseases—namely their localisation and extent—its value for determining the specific aetiology is sometimes limited. Final diagnosis within an acceptable degree of uncertainty is usually possible based on a combination of clinical, biochemical (laboratory) and imaging information. However, pathological diagnosis of lesions detected by imaging is frequently needed.

Recent advances in imaging using specific radiotracers will provide additional tools for better characterisation of a lesion at the molecular level. The combination of different functional and molecular parameters extracted from multimodality imaging techniques will probably reduce the need for pathological diagnosis of lesions.

Key examples of personalised diagnosis include:

- Patients with abdominal pain have a clinical evaluation first and — dependent on the results — additional tests including imaging are performed, which rule out important disease, confirm a clinical diagnosis or narrow a differential diagnosis.
- Patients with an acute neurologic deficit are evaluated with a brain CT and/or MRI to differentiate between ischaemic stroke, intraparenchymal haemorrhage, subarachnoid haemorrhage, sinus thrombosis or a rare cause of stroke.
- Patients with trauma are evaluated with conventional X-ray or CT scans to rule out or confirm and localise fractures, haemorrhage and contusions.
- Patients with angina pectoris are evaluated for underlying coronary artery stenosis by coronary angiography or CT angiography or for underlying myocardial viability by nuclear medicine or cardiac MRI.
- The diagnosis of diabetes mellitus or hyperthyroidism is based on laboratory examinations.
- Patients with symptoms caused by a tumour are biopsied to evaluate the type and aggressiveness of the lesion.

Diagnoses in these examples are based on clinical evaluation, imaging, laboratory examinations or evaluation of tissue specimens. Conceptually, in personalised diagnosis a large group of patients with similar symptoms is subdivided into smaller groups with a specific diagnosis and subsequent treatment allocation.

## Personalised diagnostic procedure

Medical imaging procedures are tailored to the clinical problem of the patient and to patient characteristics. The main aims are to perform the right diagnostic procedure for an individual problem, to optimise the quality of the diagnostic examination and to reduce the side effects of the diagnostic procedure. All these aims fit in the concept of PM.

Many radiological organisations provide information about the indications for imaging and the choice of modality in their referral or appropriateness criteria [6, 7]. Specific imaging protocols per modality have been designed to tackle a broad range of clinical problems. These protocols differ for example in the effective patient dose, the amount of contrast injected, injection rate, delay times, image reconstruction algorithms, contrast weightings, slice thicknesses and image orientation.

Examples of tailored protocols for a specific clinical problem are low-dose CT protocols for urinary stone detection [8], CT angiography for detection of arterial stenosis and MRI for spinal cord diseases. Examples of optimisation of a diagnostic procedure based on patient characteristics are contrast media dosing based upon patient weight [9, 10], scan delay in MR or CT angiography based on bolus tracking. Examples of patient characteristics used to reduce side effects of the diagnostic procedure are measures to avoid the use of ionising radiation when imaging children and pregnant women [11, 12], to prevent the use of IV contrast media in patients with eGFR < 30 ml/min/1.73 m² [13], and automated tube voltage selection and tube current modulation in CT based on measured attenuation [14, 15].

## Imaging in personalised prevention

Many common diseases have a long subclinical history before they become clinically evident. Biomedical imaging allows for the non-invasive assessment of in vivo structural and functional changes that may reflect specific pathologies. This makes it possible to investigate specific pathophysiological substrates of disease in the pre-symptomatic phase and at the population level. Screening a clinically healthy population with imaging to detect and characterise these subclinical abnormalities and to treat the individuals at risk might be an optimal strategy in preventive medicine. Personalised prevention with imaging will result in detection and treatment at the right time and in stratification of the main group into subgroups with low, intermediate and high risk of developing disease. Each group might have its own treatment regime, ranging from discharge, regular follow-up at several years, watchful waiting with an imaging follow-up after several months, or a medical or surgical intervention.

### Screening

Screening can be considered as PM as it can provide treatment of disease in a timely fashion before the disease has become clinically manifest with a decreased chance of implementing curative treatment. Imaging-based screening programmes are not only performed in healthy subjects. Patients can also enter an imaging screening programme to detect the progression of disease or development of cancer in an earlier phase (Table 3). The objective of these screening programmes is to start treatment earlier than it would be if based on clinical progression. Well-known examples are the image-based screening programmes for breast, lung, colorectal and prostate cancer [16–18]. Some programmes include all subjects within a certain age range; others identify high-risk categories based on simple algorithms.

**Table 3:** Examples of screening programmes with imaging

Screening programme	Image modality	Subjects	Biomarker
Breast cancer	Mammography	Females >40 years	BIRADS
Breast cancer	MRI	Females with BRCA1 or BRCA2	Lesion
Lung cancer	CT thorax	People with an increased risk profile	Nodule
Abdominal aortic aneurysm	US	Males >50 years	Maximal diameter
Hepatocellular carcinoma	US	Patients with hepatitis	Lesion
Unruptured cerebral aneurysms	DSA, CTA, MRA	Patients with small intracerebral aneurysm	Diameter (increase)
Meningioma	MRI	Patients with small meningioma	Size (increase)
Ascending aorta	CT thorax	Patients with Marfan’s disease	Diameter (increase)

The main problem with the detection of asymptomatic disease is the potential for overdiagnosis. Overdiagnosis is the diagnosis of irrelevant diseases or diseases that are sufficiently stable or indolent that they would not have become clinically relevant during the subject’s lifetime. Overdiagnosis leads to unneeded treatment with potentially harmful side effects and thus causes both economic and emotional burden. The magnitude of overdiagnosis has been recently estimated to be as much as 25 %–60 % in breast, lung and prostate cancer screening [19]. Fortunately, imaging not only plays a role in the detection of subclinical abnormalities, but can also be used to characterise the lesion as aggressive or indolent. For example, the large number of non-specific nodules detected during lung cancer screening can be addressed by follow-up imaging to assess the change in the volume of the nodule, thereby reducing the number of false-positive diagnoses [20]. A complementary molecular imaging technique allows for better characterisation of incidental findings from screening procedures, such as solitary lung nodules, dense breast lesions and suspected prostate cancer, thus avoiding unnecessary invasive procedures and biopsies in patients with negative molecular scans [21].

### Prevention of disease

Currently, prevention of cardiovascular disease and stratification of the healthy population are based on the Framingham risk score or Systematic Coronary Risk Evaluation (SCORE) score. Based on classical risk factors including age, gender, systolic blood pressure, hypertension treatment, smoking, total and HDL cholesterol, an individual is classified as having a low, intermediate or high risk. High-risk individuals are treated with intensive medical treatment while low-risk individuals are helped with lifestyle modifications. The main problem here is the large intermediate-risk group. Extensive research has been performed to better stratify patients within the intermediate risk group. As imaging is able to detect presymptomatic atherosclerotic disease, it is logical to hypothesise that imaging may become the key to identifying people who could benefit from preventive intervention. Candidate imaging biomarkers are the CT-derived calcium score from the coronary arteries [22] or more peripherally located arteries, US-derived intima-media thickness, US- or MRI-derived pulse wave velocity [23], or MRI-based plaque composition [24]. These imaging biomarkers can predict later development of disease, either on their own or by supplementing established risk factors [22]. The development of such new prevention strategies requires imaging at the population level. Population imaging is performed within the context of large population-based, prospective epidemiological studies. Population imaging focusses on finding imaging biomarkers that allow early diagnosis of preclinical disease and prediction of clinical disease. Besides imaging, information about the history of the individual subject, blood and body fluid samples, genetic data and most importantly follow-up data with regard to the development of symptoms and disease are available for correlation.

## Imaging in the selection of treatment

### Location and extent of disease

Although the question “Where is the disease and what is it?” may have been clearly answered, a clear decision on the optimal treatment cannot always be made without taking into consideration additional imaging data. For example, knowledge of the extent of a disease—a parameter of the severity of disease—is crucial for decisions on the choice of treatment. The classical example is tumour staging in which the involvement of adjacent tissues, regional lymph nodes and distant metastasis has an immediate and important impact on prognostic stratification and on the choice of therapeutic options. Improved staging with the use of imaging might help in avoiding unnecessary surgical interventions, which increases the morbidity of disease without a substantial benefit in the reduction of mortality. On the other hand upstaging can lead to the choice of more aggressive therapies, which may reduce the recurrence rate [25, 26].

Tumour staging relies strongly on cross-sectional imaging, with CT and/or MRI information being combined with quantitative metabolic information from PET and SPECT imaging. General consensus criteria and guidelines have been established based on large clinical studies combining multi-modality imaging criteria [27–29]. In these guidelines PET/CT is increasingly used in staging. This technique, which visualises specific tracer uptake in normal and abnormal tissue, allows for better localisation of cancer in structurally normal tissue. Moreover, the whole-body approach increases the sensitivity for distant metastases. The improvement in staging with PET/CT compared to PET or CT alone is substantial and has been reported for many cancers and cancers of unknown primary [30, 31]. Cost-effectiveness of preoperative PET has been proven with, for example, prevention of unnecessary surgery in one out of five patients with suspected non-small-cell lung cancer after the addition of [^18^F]FDG-PET imaging to conventional staging [32]. Recent emergence of combined PET/MR imaging systems is expected to raise the potential for combined criteria in better assessment and tissue characterisation of specific tumour and pathological lesions.

Besides oncology, cardiovascular and cerebrovascular diseases are other areas where severity of disease influences treatment decisions. Specific examples are:

- The decision to treat unruptured intracranial aneurysms is based on the location and maximum diameter of the aneurysm [33].
- Abdominal aneurysms larger than 5.5 cm are treated while smaller aneurysms are monitored during follow-up [34].
- Atherosclerotic stenosis in coronary and peripheral arteries is considered symptomatic and is treated once the diameter stenosis is more than 50 %.
- The benefit of carotid endarterectomy has been proven for symptomatic stenosis of more than 70 %. The carotid endarterectomy trials have used catheter angiography as the modality for the assessment of the severity of stenosis [35]. US, CTA and MRA have replaced this technique after extensive validation as the accurate assessment of the severity of stenosis was crucial in treatment decisions. It is therefore not surprising that carotid stenosis is one of the most evaluated imaging biomarkers in radiology.

### Tissue biopsy in interventional radiology

Final diagnosis of specific diseases and subsequent treatment allocation has been based on gross macroscopic, microscopic and/or immunohistochemical evaluation of tissue specimens. The best-known example is a tumour for which the most relevant question is the assessment of the malignant nature of the lesion and the type of malignancy. Collection of tissue samples for diagnosis can been performed by endoscopy or with a surgical procedure. US-, CT- or MRI-guided biopsy sampling is critical and most often represents the least invasive method to obtain relevant material for the in vitro diagnosis of disease in many parts of the body. Specific examples include:

- MRI-guided or contrast-enhanced transrectal US-guided prostate biopsy, which appears to be a useful method for the detection of prostate cancer in patients with prior negative biopsy results [36, 37].
- US- and MRI-guided percutaneous breast biopsy, which has replaced surgical biopsy as the initial method of diagnosis for most breast lesions [38].

Tissue sampling is not only performed for a final pathologic diagnosis, but also for the evaluation of disease spread. Here, the confirmation of potential metastasis through image-guided tissue sampling may change the type of treatment offered, for example from surgery to radiotherapy and/or chemotherapy. Specific examples include:

- US-guided core biopsy of suspicious axillary lymph nodes in patients with breast cancer allows for identification of metastatic nodes preoperatively [38, 39].
- CT-guided bone biopsy in patients with suspected bone metastasis supports a final diagnosis [40].
- US-guided biopsy in sub-centimetre neck lymph nodes improves tumour staging in head and neck cancer [41].

### Prognostic and predictive biomarkers

The essence of personalised diagnosis for personalised treatment lies in the use of biomarkers. These can be extracted from tissue, body fluids or medical imaging. In the selection of the right treatment, two different types of biomarkers are of particular importance: prognostic and predictive biomarkers. Prognostic imaging biomarkers predict the likelihood of disease progression in the absence of treatment considerations. Such biomarkers are valuable not only for identifying aggressive disease that requires immediate treatment, but also for identifying indolent disease that can safely be left alone.

- Pre-treatment tumour volume on PET/CT in breast cancer patients predicts tumour recurrence during follow-up [42].
- Baseline lesion glycolysis measured with [^18^F]FDG-PET predicts progression-free survival in diffuse large B-cell lymphoma [43].

Predictive biomarkers indicate the relative sensitivity or resistance to drug treatment or the success of treatment by surgery, interventional radiology or radiotherapy.

- CT-derived perfusion parameters in metastatic renal cell cancer can predict the biological response to antiangiogenic drugs [44].
- Dynamic contrast-enhanced (DCE) MRI to evaluate the non-enhancing tumour fraction predicts the response to chemo-radiotherapy in advanced cervical cancer [45].

Both types of biomarkers are relevant in PM as they influence the decision for the treatment, type, length and intensity of treatment. A specific application of a prognostic or predictive biomarker is the monitoring of the response after the first cycle of treatment (chemotherapy and/or radiotherapy) in order to predict the final response, progression-free survival, recurrence and overall survival.

- [^18^F]FDG-PET response after the first cycles of chemotherapy predicts the final response as well as progression-free survival and overall survival [46].
- Diffusion-weighted (DWI) MRI of metastatic ovarian and peritoneal cancer was not able to predict response, while an increase in ADC after the first or third of six cycles of chemotherapy was able to predict tumour response [47].

Multiple prognostic and predictive imaging biomarkers have been identified. The main challenge for PM is the translation of this information in patient management pathways and treatment algorithms.

### Molecular imaging

PM implies the identification of the specific genomic and molecular substrate of alterations leading to disease and “medical treatment tailored not just to symptoms, but to the biochemical profile of an individual’s disease state (gene expression, proteome, dominant metabolic pathways, etc.)” [48]. This has tremendously changed the evaluation of diseased tissue specimens: from microscopic and/or immunohistochemical evaluation to gene expression profiling.

Parallel to this development, medical imaging has changed. Traditionally, medical imaging has been a tool for non-invasive assessment of anatomy and for detection and localisation of disease processes. Currently, a wide variety of new medical imaging techniques produces important information about physiology, metabolism, molecular biological processes and functional genomics. Multiple methods have been described for imaging the temporal and spatial biodistribution of a molecular probe as well as related biological processes such as cell proliferation, apoptosis, angiogenesis, hypoxia, and gene activation and expression. These new methods combine the ability to measure and quantify biological processes with the ability to localise the measured entities onto a high-resolution anatomical and functional image [49]. In the future, an imaging diagnosis will not only include the location and extent of diseased lesions but also information on the physiological characteristics and biological behaviour of the lesion (e.g., perfusion, flow, metabolism, diffusion).

Molecular imaging is likely to replace several aspects of ex vivo tissue analysis, which will be a key component in PM. It is expected that molecular imaging can be used to detect diseases much earlier because molecular and cellular changes precede anatomical and structural changes on classical imaging modalities such as CT and MRI. As such, it will contribute to PM by treating the patient at the right time. As new therapies will be increasingly based on a foundation of precise understanding of the molecular basis of disease, simultaneous creative probe development will require a deeper understanding of cancer biology [50].

- Hypoxia is common in malignant tumours and increases the resistance to radiation therapy. [^18^F]-fluoromixonidazole ([^18^F]-MISO) imaging enables the non-invasive assessment of tumour hypoxia, and its use to predict response and survival benefits from treatment has been demonstrated in oesophageal cancer, lung cancer and renal cell carcinoma [51–53].
- Molecular imaging may help improve staging of malignancies. In a preclinical animal model, detection of prostate cancer and metastasis was possible with the use of an imaging reporter gene under the control of the promoter of astrocyte elevated gene-1 (AEG-1), which is selectively active only in malignant cells. Metastatic lesions were identified through bioluminescence imaging and SPECT with a higher sensitivity than [^18^F]FDG and [^18^F]-NaF [54].

### Biology-based tissue biopsy in interventional radiology

PM currently requires an in-depth analysis of the genetic expression and molecular and cellular biology of the individual disease. This is based on a more refined evaluation of tissue samples by assessment of molecular and genetic expression patterns. For example, for malignant tumours it is has become relevant to know which of the genetic alterations is functionally relevant for abnormal cell proliferation.

Recent studies have shown that malignant lesions demonstrate intratumoral heterogeneity with regard to genetic alterations and expression patterns, thus indicating the existence of multiple subpopulations of cells. Furthermore, metastatic lesions may have genetic expression patterns that differ from the expression pattern in the primary lesion or from each other, and these lesions can therefore show a different behaviour and response to treatment. For more effective targeted therapy, it is therefore necessary to identify these mutations and confirm them by individual tissue analysis of each subset.

The differences in genetic expression patterns are frequently reflected as differences in imaging appearance. Imaging can thus further play a crucial role in guiding the sampling procedure. For example:

- Contrast-enhanced US in liver tumours not only increases the accuracy of biopsies by improving the visualisation of poorly visible lesions but also helps target the biopsy to the hypervascular areas of a given lesion [55].
- [^18^F]FDG or [^11^C]-methionine-PET can detect hypermetabolic areas in brain tumours and improve the diagnostic yield of stereotactic brain biopsy [56].
- PET-directed, US-guided biopsies with a PET/US fusion targeted biopsy system improve prostate cancer detection [57].

### Theranostics/companion diagnostics

One of the classical examples of PM is the determination of the status of the human epidermal growth factor receptor 2 (HER2) expression in patients with breast cancer as a prerequisite for treatment with trastuzumab (Herceptin, Genentech). In some cancers, HER2 is over-expressed, causing cancer cells to reproduce uncontrollably [58]. Trastuzumab is a monoclonal antibody that interferes with the HER2 receptor and inhibits the effects of overexpression of HER2. This approach targets one of the core deregulated cellular signalling pathways in cancer, which is totally different from cytotoxic chemotherapy. If the breast cancer does not overexpress HER2, trastuzumab will have no beneficial effect. The coupling of therapy with diagnostic information specific for the intended target is called a theranostic system.

In the HER2-trastuzumab theranostic system, the diagnostic test is a laboratory test on a tissue specimen in vitro, but in vivo imaging can also provide diagnostic tests for theranostic systems. The most common example is ^123^I/^131^I for targeted radionuclide radiotherapy in differentiated thyroid cancer. Targeted radionuclide therapy combines “the favourable targeting properties of peptides and antibodies with the effectiveness of radiation-induced cell death. A major advantage is the possibility to determine the selective accumulation in the targeted tissue by molecular imaging studies using structurally identical diagnostic compounds” [59, 60].

Theranostics relying on in vitro testing of small tumour samples does not address the problem of tumour heterogeneity or the phenotypic dedifferentiation of tumour metastases. By using different molecular imaging tracers, we begin to appreciate the heterogeneity of metastatic disease within individual patients. Tumour heterogeneity can often be seen even within the same lesion. Thus, in a patient with metastases of differing biology, a single therapy approach may result in a mixed tumour response. It is not possible to biopsy each and every lesion, so molecular imaging is essential to successfully tailor treatment to the individual tumour biology. A theranostic concept that uses molecular imaging will better address the complexity of tumour biology and will therefore have the potential to enable improved diagnosis, treatment, and monitoring of treatment response all at once [50].

Theranostics provide a unique signature for improved staging of the tumour, for imaging of the biodistribution of the target to predict the biodistribution of the radiation dose and to individually monitor the efficacy of treatment with the same basic compound that is being used to target and treat the disease [60]. Furthermore, the development of new specific biomarkers that can be used for both in-vivo diagnostic imaging and targeted therapy with alpha emitter compounds or carriers of cytotoxic agents will further expand the scope of the application of theranostics in clinical practice. It is even foreseeable that advanced targeted therapy, which is often extremely expensive, would only be applicable if we have a companion diagnostic biomarker that can predict the efficacy of the treatment.

Aside from the targeted therapy with ^131^I, other examples of image-based theranostics are:

- The norepinephrine analogue metaiodobenzylguanidine (MIBG) conjugated to ^131^I. MIBG is used for diagnostic purposes, and it is available for targeted therapy of neuroendocrine tumours in adults and neuroblastoma in paediatric patients [61, 62].
- Somatostatin peptide analogues are used in both imaging and radionuclide-based therapy for endocrine tumours [63].
- Numerous other examples of paired molecular imaging therapy are found with other radiopharmaceuticals that are selective for biochemical processes, such as cellular proliferation, steroid synthesis, growth factor receptor expression, catecholamine production, hypoxia-induced gene expression or apoptosis [64–66].

### Nanotechnology

The evolving era of nanotechnology will further boost the development and use of theranostics. Nanoparticle-based imaging and therapeutic agents are a specific, advanced and promising form of theranostics. Nanoparticles are synthesised from organic or inorganic materials such as lipids, polymers, metals or semiconductors. Thereafter ligands have to be conjugated to the nanoparticle in order to target cellular receptors (e.g. overexpressed in tumour-induced angiogenesis) or other molecular characteristics of diseased tissue [64]. In addition, visualisation is achieved by conjugation of imaging agents such as radionuclides or paramagnetic/superparamagnetic metals. If the diagnostic scan demonstrates that the lesion is positive for the target, the patient can be treated with the same nanoparticle, which now carries the therapeutic agent. The therapeutic agent can be cytotoxic radionuclides or drugs encapsulated in the nanoparticle structure. In the former case, the nanoparticles have to be engineered with a moiety for chelation of radiometals. Other materials that can be delivered locally are interfering RNAs, genes and peptides. A major advantage of nanoparticles for theranostics would be the high payload of both the therapeutic agent and imaging probe that can be delivered to the target tissue. Specific examples of theranostic agents currently under investigation include:

- liposomal vesicle formulations containing cytostatic drugs and MR contrast agents [67, 68].
- carbon nanotubes as multifunctional carriers for radioisotopes for treatment and imaging [69].

### Radiogenomics/integrated diagnostics

Radiogenomics is a new approach in which large sets of complex descriptors of disease are extracted from routine clinical images and related to molecular biology and gene expression patterns of disease [70, 71]. It is based on the assumption that the observed image is the product of mechanisms occurring at a genetic and molecular level. Thus, parameters extracted through image processing and analysis are linked to the genotypic and phenotypic characteristics of the tissue [72].

Like other ‘omics techniques, it is not hypothesis-oriented but goal-oriented. In radiogenomics, an a priori hypothesis is not formulated on which imaging technique or feature should be used to reflect a certain biological mechanism. Every imaginable feature is extracted from an image data set, including semantic descriptors (e.g., shape, spiculated patterns, presence of necrosis, localisation) and/or quantitative features. These features are then correlated to gene expression profiles. Radiogenomics is the term used when imaging features are correlated to gene expression. The very large numbers of correlated parameters (usually over a hundred, ideally several hundreds) extracted from the same data sets requires specific statistical methods similar to those used in other ‘omics.

One of the first radiogenomics publications linked CT imaging features to gene expression in hepatocellular carcinoma [73]. The expression variation of over 6,000 genes was grouped into 116 gene modules. By combining 28 imaging traits into similar modules, 78 % of gene expression profiles could be predicted. This means that the relative expression of a given gene could be determined in any given liver tumour. This approach has been successfully applied to MRI features of glioblastoma multiforme and gene expression profiles, protein expression [74, 75], microRNAs [75], messenger RNA expression and DNA copy number variation [76]; to MRI features of breast cancer and gene expression profiles [77]; to CT features of renal cell carcinoma and gene mutations [78] and to [^18^F]FDG features of lung cancer and protein expression [79].

It is clear that medical images inherently contain a wealth of mineable information, which reflects genetic and molecular information of disease in an individual patient. This would potentially allow particular imaging phenotypes to serve as surrogates for the unique molecular programmes that typify a molecular subtype of cancer [80]. Radiogenomics will definitely contribute to personalisation of patient management and help improve existing staging and diagnostic schemes. Imaging can be used to determine the molecular diversity of disease and thereby stratify patients into molecular subclasses with different prognoses [72]. For example, one study demonstrated that imaging features of hepatocellular carcinoma were predictive of a gene expression profile of venous invasion genes and subsequently predictive of a poor outcome [73]. Equally important is the use of imaging to identify patients for whom targeted therapeutic agents will be effective. For example, one study demonstrated that specific imaging traits in GBM were associated with an EGFR protein expression programme and EGFR protein expression [74]. Finally, intratumoral genetic heterogeneity can be reflected in intratumoral imaging heterogeneity, which demonstrates one advantage of imaging over tissue analysis.

Although PM has been boosted by the huge developments in molecular profiling, it is clear that nowadays imaging can provide comparable information. However, competition between the two approaches is not foreseen as each acts on a different level. Michael Kuo, one of the pioneers in radiogenomics, sees a blurring of the traditional lines between the diagnostic sciences and a merging of molecular diagnostics and imaging into a single diagnostic discipline [80]. Integrated diagnostics must include multimodality and multiparametric imaging approaches as well as multiple pathologic, molecular and genetic assays. This integration of the huge diagnostic data from all levels—from genes, molecules, cells, tissues, organs and organ systems—is needed in the era of PM.

## Evaluation of treatment response

Imaging plays an important role in the assessment of therapeutic response. Classically decreased morbidity, e.g. relief of symptoms, and mortality have been endpoints for treatment evaluation. In most diseases treatment response can be evaluated more objectively, reproducibly and earlier with imaging than by monitoring symptoms. An early and accurate therapeutic response evaluation is critical in PM as it can influence the decision on discontinuation of treatment, treatment adjustment and/or additional treatment. Imaging can spare patients from prolonged exposure to ineffective treatments and allow alternative therapies to be applied sooner. Imaging not only plays a role in assessing response to chemotherapy, radiotherapy and/or targeted therapies, but also in the monitoring of changes after image-guided intervention.

Timing of response evaluation can differ according to clinical needs. To prevent aggressive treatment — which could be accompanied by severe side effects or expensive treatment in non-responders — it is crucial to evaluate early response in order to discontinue treatment and consider a second-line treatment that is potentially more effective. However, final treatment response evaluation is needed to decide on subsequent surgical treatment or neo-adjuvant treatment after presurgical chemoradiotherapy.

Crucial in the evaluation of treatment is the assumption that early tumour response is related to the final treatment response and that both early and final treatment responses are related to overall survival and/or progression-free survival. Multiple studies have already been performed that demonstrated these relationships. It is now time to integrate and translate these findings into clinical trial design and clinical guidelines, which describe the clinical decisions based on the results of imaging tests. This is especially the case with the further expansion of cancer management based on PET imaging. For example:

- In colorectal cancer, PET of the liver could predict tumour response to chemotherapy in liver metastasis, and PET of the pelvis during or after (chemo)radiotherapy could predict the final tumour response and overall survival [46].
- Patients with advanced oesophageal cancer and no evidence of response to neo-adjuvant chemoradiotherapy on PET imaging, which occurs in 50 % of the patients, have a worse prognosis after surgical resection than patients who respond, even after radical surgical resection [81].
- For both limited stage and advanced stage Hodgkin lymphoma, multiple studies have demonstrated that interim PET imaging has a high negative predictive value and a moderate positive predictive value for progression-free survival. Multiple trials are evaluating the effect of treatment adaption based on early PET evaluation on progression-free survival. These studies will determine whether therapy can be safely escalated or reduced based on treatment response evaluation [31].

### How to assess treatment response?

Treatment monitoring in malignant tumours is based mainly on anatomical imaging, frequently still assessed on 2D images, e.g., according to the RECIST criteria. These criteria have been expanded (RECIST 1.1) to include lymph node evaluation and the use of PET [82]. Anatomical imaging detects morphologic changes, which occur relatively late during treatment. Over the last decade, some shortcomings of anatomical imaging with regards to therapy response have become evident [83]. These relate to a poor reproducibility of tumour measurements [84, 85] and inconsistencies between tumour response and survival [86]. Anatomical imaging alone does not support a comprehensive understanding of tumour biology. Furthermore, anatomical imaging falls short of fully assessing the response to targeted therapies that exert a cytostatic rather than a cytotoxic effect as well as the early response assessment to neoadjuvant systemic treatments.

The field of treatment response evaluation is a prime example of the gap between current practice and advances in imaging and image analysis techniques. Three-dimensional display of lesions and volumetric measurements have advanced and may yield advantages in the assessment of tumour response. For example, comparison of both single-diameter and volumetric measurements to assess treatment response demonstrated significant differences in tumour response [87, 88]. In addition, volumetric measurements for response assessment provide more reproducible results [89]. Advances in quantitative imaging and image analysis will contribute to changing the landscape of clinical practice and clinical trials [90, 91].

The development of new imaging techniques such as molecular, metabolic and functional imaging has paralleled the expanding knowledge on tumour biology. These techniques provide additional critical information for care of individual patients when used as a supplement to anatomic imaging, as they expand the ability to biologically characterise tumours and monitor tumour response at a more appropriate level. Cancer is currently seen as a dysregulation of a restricted number of signalling pathways, and treatments that target these pathways will lead to successful therapies. Molecular imaging helps assess changes in the tumour that would indicate the activity of one or more of the key dysregulated pathways. For example, for quantitative analysis of tumour response, [^18^F]FDG-PET imaging — which assesses the glucose uptake and thus the metabolic activity level of various cancers — has been identified as a reliable indicator of treatment response. PET-based scoring criteria extend the morphological RECIST criteria into a biological dimension with a proposed PERSIST score [92]. This approach was found to be more consistent and showed better prediction of patient response to the treatment in a variety of cancers. A wide range of more specific probes and new imaging biomarkers are currently emerging for assessment of individual types of cancer and monitoring response to treatment.

Advanced imaging can also target the problems related to the use of one single marker for treatment response evaluation. While intratumoral heterogeneity explains the individual response to (targeted) therapy, a monitoring approach based on a single marker is not sensitive to non-response in a heterogeneous lesion or in one of multiple lesions. “This defines the weakness of serum analyses, which provide an average signal of output from all lesions, or of a biopsy program to characterize a tumour—if all can be different, then all must be characterized” [93]. Accurate localisation of regional differences in tumour behaviour and tissue characteristics is the strength of imaging. But even a molecular imaging approach based on a single probe may be insufficient, as alternative dysregulated signal pathways may replace the function of the targeted pathway [93, 94]. Currently, imaging can be performed with different modalities integrated into hybrid systems (SPECT/CT, PET/CT or PET/MRI) and can cover several functional levels. Multi-parametric imaging can potentially better identify heterogeneous and localised response to treatment. By combining imaging markers from different pathways, it may be possible to assess the type of physiological escape mechanisms of a tumour (or other disease) [94, 95], enabling individual treatment to be fitted in real time to the type of response which is, of course, PM in its most basic sense.

- For example, [^15^O]-labelled water PET to assess blood perfusion and [^18^F]FDG-PET to assess glucose metabolism was simultaneously applied in females with locally advanced breast cancer treated with neoadjuvant chemotherapy. Changes in PET measures of tumour blood flow and glucose metabolism predicted disease-free survival and overall survival [96].
- Similar evaluation criteria could be expected by combining functional parameters of diffusion and perfusion images combined with metabolic PET data obtained using hybrid PET/MR systems.

### Imaging in drug discovery and drug trials

Treatment response is relevant for clinical practice but equally important for pharmacological research, where it can be used to identify and evaluate novel drugs. Indeed, in its Critical Path Initiative, the US Food and Drug Administration (FDA) has recognised the new role of medical imaging in drug development and regulatory approval [97]. Although the Initiative emphasises the role of imaging in the assessment of biomarkers, medical imaging also has other applications in drug discovery: “a molecular imaging probe can be used to determine target occupancy before and after administration of a new drug candidate, which helps to assess binding affinity and to determine correct dosages” [98].

In pharmaceutical research, the demonstration of tumour response is an important element of early drug development. Further investigation is often not performed when antitumour activity and treatment response are not demonstrated. Anatomical imaging is limited to revealing a physical shrinkage of the tumour. With the current molecular and functional imaging techniques all kinds of antitumour activity can be detected such as changes in receptor expression and tumour perfusion. It is expected that these new techniques, which integrate knowledge on the tumour biology, will foster the detection of relevant targeted drugs.

Very important is the increasing use of (imaging) biomarkers as surrogate endpoints for clinical trials. Given that validated surrogate endpoints allow dramatic shortening of clinical trials by shortening the time to the study end point, with associated savings and a faster availability of new treatments, this is an area in which both the pharmaceutical industry and healthcare authorities are pushing for biomarker use. Molecular imaging and radiopharmaceuticals are particularly important within this context as they allow in vivo visualisation of the effect of the treatment.

## Personalised treatment

Each intervention on a patient has a personalised approach. This is true for surgery, radiotherapy and interventional radiology. The same disease is treated differently based on disease and patient characteristics provided by imaging before and during the intervention. Imaging helps to plan and guide surgical or minimally invasive individualised therapies. For example, mapping of the anatomy before and during liver surgery is based on imaging. Stereotactic brain biopsy is guided by imaging. Surgical planning of brain tumours is based on fMRI, which maps eloquent areas, and DTI, which visualises relevant brain tracts [99]. In case of complications during an intervention, the solution is even more individualised.

### Radiotherapy

Imaging has always been involved in radiotherapy through image-guided radiation therapy planning (RTP), including the verification of treatment delivery. Delineation of primary and metastatic lesions, which helps to optimise target volume definition in radiotherapy, is based on imaging. Initially, the introduction of cross-sectional imaging has tremendously improved the field of radiation oncology [100]. Intensity-modulated radiotherapy, which improves dose delivery to the tumour while reducing exposure of healthy tissue to limit the side effects of treatment, requires a precise delineation of the tumour. For that reason MRI is increasingly used in treatment planning as it provides better soft tissue contrast, which improves target volume definition. A next step is the application of functional imaging techniques, which can provide information on more aggressive parts or more radiation-resistant parts of the tumour. The target volume definition is then based on the biology of the tumour.

- Hypoxia imaging with [^18^F]MISO-PET in head and neck cancer can detect regions that are resistant to treatment. Combined [^18^F]MISO-PET imaging and IMRT planning permit dose painting with delivery of much higher doses to hypoxic regions [101].

Some tumours are located in areas of the body that are prone to movement, such as the lungs, liver and prostate gland, and some tumours are located close to critical organs and tissues. In these patients, image-guided radiation therapy (IGRT) is used in which imaging immediately before or even during a course of radiation therapy is applied to improve the precision and accuracy of the delivery of treatment. In addition, another application of IGRT is the reduction of the target volume during the course of radiation treatment based on the visualisation of tumour shrinkage with imaging.

### Interventional Radiology

Image-guided interventions provide the means to deliver therapies locally at the disease site, whether the therapies are based on chemical compounds (including drugs and radiotherapeutics), genes, devices (including stents), cells, sound waves (high-intensity focused ultrasound = HIFU) or induction of ablative temperatures (using hyper- and hypothermia with radiofrequency, microwaves or cryotherapy). Most current interventional image-guided procedures are individually tailored to the local anatomic and functional circumstances as well as the personal needs of the patient. In this way, image-guided interventions are in themselves an important and integral part of PM. For example:

- Embolisation of bronchial arteries in patients with haemoptysis is guided by CT angiography and DSA during interventions.
- Decisions about the treatment of HCC depend on the location, size and number of tumours at each time of presentation, favouring ablation for limited disease and (chemo)embolic techniques for more extensive spread.

Medical imaging is going to play an important role in individualised drug delivery. In the past 30 years, drug delivery research was focussed on targeting carriers such as liposomes, polymeric nanoparticles and micelles to the disease site. New developments now promote the selective release of bioactive compounds after application of a trigger [102]. The trigger mechanism can be applied locally and at specific times, depending on the individual patient’s needs, and can be selectively applied with interventional techniques such as HIFU under the guidance of MRI [102]. Although for many reasons most tumour ablations are performed using percutaneous radiofrequency or microwaves, HIFU is the only clinically viable technology that can be used to achieve a local temperature increase deep inside the human body in a non-invasive way. MRI can be used to provide continuous temperature mapping during HIFU for spatial and temporal control of the heating procedure and prediction of the final lesion based on the received thermal dose [103].

## Conclusions and recommendations

The future of medicine is in early diagnosis and individually tailored treatments, a concept that has been designated as ‘personalised medicine’ (PM), which relates to delivering the right treatment to the right patient at the right time. For PM to reach its full potential, medical imaging must be an integral part.

Imaging procedures are tailored to the clinical problem and patient characteristics. Screening for preclinical disease is done with imaging. Stratification based on imaging biomarkers can help identify individuals suited for preventive intervention. Treatment decisions are based on the in vivo visualisation of the location and extent of an abnormality as well as the loco-regional physiological, biochemical and biological processes using structural and molecular imaging. Image-guided biopsy provides relevant tissue specimens for genetic/molecular characterisation. In addition, radiogenomics relate imaging biomarkers to these genetic and molecular features. Furthermore, imaging is essential to patient-tailored therapy planning, therapy monitoring and follow-up of disease, as well as targeting non-invasive or minimally invasive treatments, especially with the rise of theranostics. Radiologists need to be prepared for this new paradigm as it will mean changes in training, clinical practice and research.

In order for radiologists to take on these new challenges they must be involved in the following activities:

- Promotion of the use of non-invasive medical imaging as part of PM.
- Integration of anatomical, functional and molecular imaging in one imaging department.
- Validation of quantitative imaging biomarkers for diagnosis and treatment repsonse assessment.
- Implementation of image-guided interventional procedures for providing the most accurate tissue samples for pathology-based biomarkers.
- Integration of personalised diagnostic imaging and therapeutic procedures (theranostics).
- Provision of personalised image-based phenotypic results that complement genomic analysis (radiogenomics).
- Esthablishment of large imaging biobanks that are linked to other -omics data.
- Delivering personalised treatment via a wide range of interventional radiologic procedures.
